# Death Domain-Associated Protein Promotes Colon Cancer Metastasis through Direct Interaction with ZEB1

**DOI:** 10.7150/jca.34233

**Published:** 2020-01-01

**Authors:** Yanliang Liu, Fengqin Guo, Xu Zhu, Wenyi Guo, Tao Fu, Weixing Wang

**Affiliations:** 1 Department of Gastrointestinal Surgery II, Key Laboratory of Hubei Province for Digestive System Disease, Renmin Hospital of Wuhan University, Wuhan, Hubei Province, China; 2 Department of Gynaecology and obstetrics II, Key Laboratory of Hubei Province for Digestive System Disease, Renmin Hospital, Wuhan University, Wuhan, Hubei Province, China; 3 Department of Hepatobiliary Surgery, Renmin Hospital of Wuhan University, Wuhan, Hubei Province, China

**Keywords:** colorectal cancer, DAXX, E-cadherin, metastasis, ZEB1

## Abstract

**Background:** Death domain-associated protein (DAXX) is a tumor suppressor and its loss has been found in a variety of cancer types. Dysregulation of DAXX is strongly correlated with cancer metastasis. However, the role and functions of DAXX in colorectal cancer (CRC) metastasis are not fully understood.

**Methods:** We validated the mRNA and protein expression of DAXX in CRC specimens and CRC cell lines using real-time reverse transcription-PCR and Western blot, respectively. The overexpression plasmids of ZEB1 and E-cadherin and the siRNAs for DAXX and ZEB1 knockdown were constructed to study the impact of these factors on cells. Wound-healing assay and Transwell assay were performed to examine the cell motility and cell migration and invasion abilities, respectively. Luciferase assay was performed to assess the E-cadherin promoter activity. Immunoprecipitation assay was performed to investigate the interaction between proteins. The rescue experiment was carried out to verify whether the effect of DAXX on E-cadherin expression is depended on ZEB1.

**Results:** DAXX expression was lower in liver metastases than in primary colon cancer tissues. Our results demonstrated that DAXX directly interacted with ZEB1 and suppressed its inhibitory effect on promoter activity of E-cadherin through a ZEB1-dependent manner, and thus suppresses the cell motility, migration, and invasion of CRC cell lines.

**Conclusion:** In sum, these findings supported that the loss of DAXX is associated with cancer cell metastases in CRC. ZEB1-mediated transcriptional suppression of E-cadherin is a possible mechanism. DAXX/ZEB-1 pathway could be a potential therapeutic target for preventing cancer metastasis in CRC.

## Introduction

Despite improvements in diagnosis and therapy over the last decade, colorectal cancer (CRC) is still a major cause of cancer-related death worldwide, accounting for over 800 thousand deaths each year 1. The five-year survival rate of metastatic CRC patients is extremely dismal 2. Systemic therapy for late-stage CRC typically includes 5-FU, cisplatin, or doxorubicin 3-5, but resistance usually develops after treatment. Metastasis is a well-known factor of poor prognosis 6. Understanding the mechanisms of CRC metastasis can help identify new molecular targets for the development of novel therapies and improve the clinical outcome.

The tumor suppressor gene: death domain-associated protein (DAXX) is down-regulated in various cancers, such as lung cancer and CRC 7. Evidence has shown that dysregulation of DAXX is strongly correlated with metastasis. Further, previous studies suggested that DAXX may play different roles based on its subcellular location—in the nucleus or in the cytoplasm 8. In the cytoplasm, DAXX appears to be associated with apoptosis. However, in the nucleus, DAXX can directly bind to many transcription factors and serve as a potent transcriptional repressor that affects tumor development and progression 7, 9, 10. Lin C-W et al. have reported that DAXX can inhibit metastasis by directly interacting with HDAC1 to attenuate the Slug axis in lung cancer 11. In prostate cancer, DAXX has been demonstrated to act as a negative androgen receptor (AR) partner and inhibits AR-mediated promoter activity through direct protein-protein interactions 12. Overexpression of DAXX induces G1 cell cycle arrest through direct binding to transcription factor 4 (Tcf4), affecting the expression of genes downstream of Tcf4 13. In CRC, DAXX expression in cancer tissues is lower than that in normal colon specimens 15. Despite the finding that DAXX is underexpressed in CRC, its role and functions in CRC metastasis have not been systematically explored.

The vertebrate zinc finger E-box binding homeobox (ZEB) proteins are a family of transcription factors that mediate gene expression in physiological and pathological processes 14. In particular, the ZEB proteins play a key role in tumor progression and metastasis. Evidence has shown that ZEB proteins may act as oncogenic proteins in several cancers and are up-regulated during malignant transformation 15. Some studies have reported that ZEB proteins are involved in a variety of signaling pathways in several cancer types and are strongly correlated with a poor clinical outcome. For instance, in hepatocellular carcinoma, researchers have found that ZEB1 up-regulation is associated with advanced TNM stage, metastasis, and frequent, early recurrence 16, 17. In pancreatic cancer, ZEB1 and ZEB2 cooperate in both the epithelial-mesenchymal transition (EMT) and the mesenchymal-epithelial transition processes 18. An important mechanism of ZEB-mediated cancer cell migration and invasion is through the regulation of E-cadherin 19. E-cadherin acts as an adhesion molecule that impacts cell dissociation and detachment and thus suppresses cell motility, migration, and invasion 20. However, the regulation of E-cadherin remains poorly understood. In various cancer types, studies have shown that ZEB proteins can directly bind to the promoter of E-cadherin and suppress its expression at the transcriptional level 21. Further, studies have shown that DAXX can markedly inhibit several transcription factor-responsive reporters, including E2F1, Sp1, and NF-κB 10, but the mechanisms of such regulation have not been clearly demonstrated. Given the critical roles for DAXX, E-cadherin, and the ZEB proteins in cancer development and metastasis, we sought to investigate how DAXX regulates E-cadherin in the metastasis of CRC and how the ZEB proteins are involved in the process.

## Materials and Methods

### Cell culture and plasmid construction

293T, SW48, HT29, LoVo, Caco2, HCT116, and SW620 cell lines were purchased from the American Type Culture Collection. Cells were cultured in high glucose-DMEM supplemented with 10% fetal bovine serum (FBS; Gibco, ThermoFisher, MA, USA) with 5% CO_2_ at 37℃. A fragment of the E-cadherin coding sequence was inserted into the pCDNA3.1 vector. The promoter of E-cadherin was inserted into the pGL6-basic luciferase vector (Promega, WI, USA).

### Western blot

Briefly, cells were lysed by RIPA buffer (150mM NaCl, 0.5% EDTA, 50mM Tris, 0.5% NP40), then protein lysates (15-50μg) were loaded and separated on the 10% sodium dodecyl sulfate-polyacrylamide gradient gel. Next, the proteins were transferred onto PVDF membranes and corresponding bands were incubated with the primary antibody and horseradish peroxidase-conjugated secondary antibody. Those bands were then analyzed using the ECL chemiluminescence system (Pierce, IL, USA).

### siRNA and plasmids transfection

To silence endogenous DAXX and ZEB1, the target sequences were purchased from RIBO company (Guangzhou, China). Cells were transiently transfected with siRNA using Lipofectamine 2000 transfection reagent (Life Technologies, ThermoFisher, MA, USA) for 48-72h according to the manufacturer's instructions. For overexpression experiments, cells were transfected with 5ng plasmids by Lipofectamine 2000 transfection reagent according to the manufacturer's instructions.

### Wound-healing assay

Briefly, approximately 50×10^6^cells were seeded in a 6-well plate for 24h. Then, a wound was carefully scrapped with a 10μl sterilized pipette tip. The coverage of the scratched area was examined at different positions and the wound widths were measured by ImageJ software (National Institutes of Health, MD, USA). All experiments were conducted in at least triplicates.

### Luciferase assay

The 293T cells were plated in 6-well plates and then co-transfected with siRNA and/or E-cadherin promoter-luciferase plasmids (pGL6) according to the manufacturer's instructions. After 48h, luciferase activity was measured using a Dual-Luciferase Assay kit (Promega, WI, USA). All experiments were conducted in at least triplicates.

### Cell migration and invasion assays

Cell migration and invasion assays were employed according to previous reports 23. For migration assay, 4×10^4^cells were incubated in 200μl serum-free medium and were seeded into the upper chamber, 500μl DMEM with 10% FBS was added to the lower chamber of Boyden chamber (Sigma, MO, USA). Cells were incubated for 16h according to the manufacturer's instructions. Finally, membranes were fixed in 4% paraformaldehyde for 15min at room temperature and were stained with 2% crystal violet for 20min. The number of cells was observed and images were obtained under a microscope (100 × magnification). For invasion assay, the membrane was pre-coated with 50μl diluted Matrigel and all other steps were the same as the migration assay.

### RNA extraction and Real-time RT-PCR

Total RNA was extracted from cells using Trizol reagent according to manufacturer's instructions and cDNA was synthesized using a RevertAid^TM^ First Strand cDNA Synthesis Kit (ThermoFisher, MA, USA). Then, real-time reverse transcription-PCR (qPCR) was performed with SYBR Green SuperMix (TransGen Biotech, Beijing, China). The relative mRNA expressions were normalized to GAPDH using the 2^-ΔΔCT^ method.

### Immunofluorescence

Cells were plated into 24-well plates covered with glass. Cells were fixed with 4% paraformaldehyde and permeabilized with 0.1% Triton X-100. Then, cells were blocked with 1% bovine serum albumin and incubated with primary antibody. Next, cover slips were incubated with corresponding fluorescein second antibody for 1h and nuclei were stained with DAPI. Images were obtained using FV1000 confocal laser-scanning microscope (Olympus, WI, USA).

### Patient specimens and materials

Patient specimens were obtained from Renmin Hospital of Wuhan University. Patients were enrolled in this study according to the following criteria: 1) confirmed CRC by three independent pathologists on tissue biopsy; 2) expected to undergo surgery; and 3) had not received radiotherapy and/or chemotherapy before surgery. Cancer tissues obtained during surgery were collected and stored into liquid nitrogen. All patients signed informed consent prior to enrollment in this study. The study was approved by the Institutional Review Board of Renmin Hospital of Wuhan University and conducted according to the Declaration of Helsinki.

### Statistical analysis

All experiments were conducted in at least triplicates. Statistical analysis was performed with SPSS 18.0 (SPSS Inc., IL, USA). Mean±standard deviation (SD) was calculated for each variable and Student's *t*-tests were conducted between different groups. *P*<0.05 was considered statistically significant.

## Results

### DAXX is underexpressed in metastatic CRC specimens and CRC cell lines

**Figure 1:**
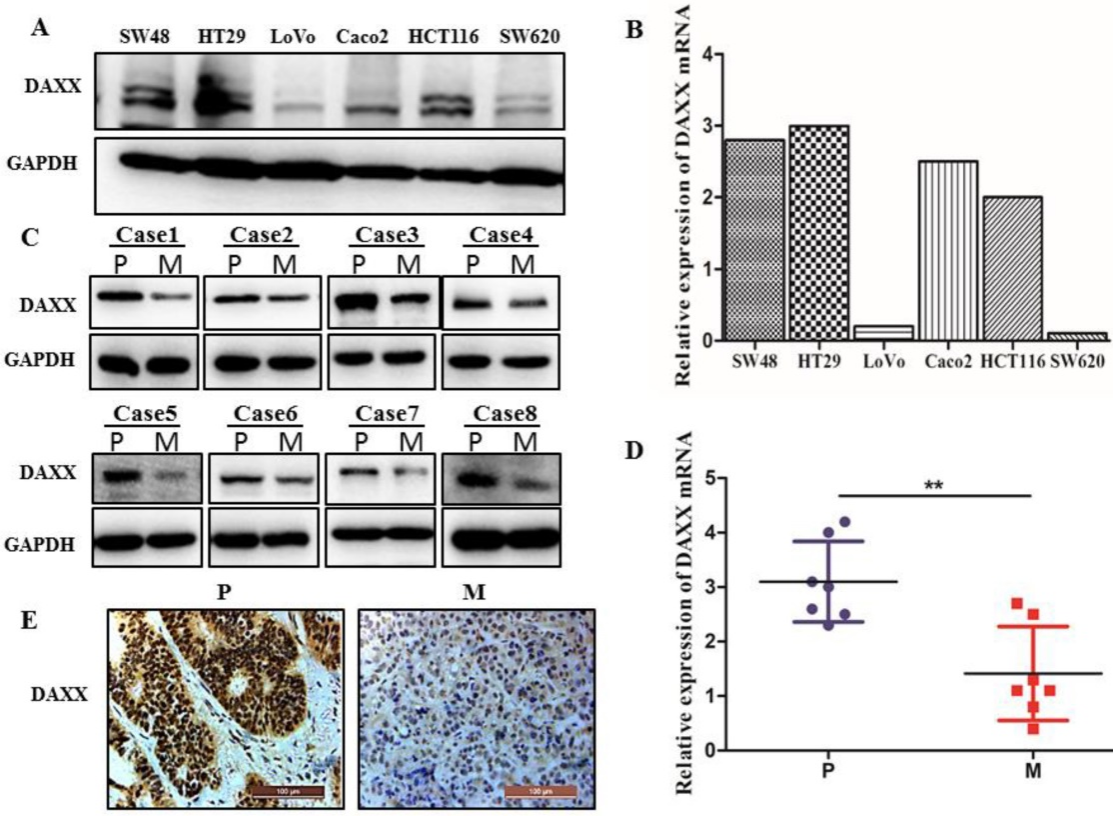
** The mRNA and protein expression of DAXX in metastatic CRC specimens and CRC cell lines. (A)** Protein expression of DAXX in different CRC cell lines by Western blot. **(B)** mRNA expression of DAXX in different CRC cell lines by qPCR. **(C)** Protein expression of DAXX in primary colon cancer tissues and metastasized liver tissues by Western blot (P: primary colon cancer tissue; M: metastasized liver tissues). **(D)** mRNA expression of DAXX in primary colon cancer tissues and metastasized liver tissues by qPCR (**P<0.05). **(E)** Immunohistochemistry results demonstrated that the DAXX level was lower in the metastatic sites than in the primary tumors (Scale bar: 50μm).

We first examined the level of DAXX mRNA and protein by qPCR and Western blot in different CRC cell lines. As shown in Figure 1A and B, DAXX mRNA and protein levels were markedly lower in metastatic CRC cell lines than in non-metastatic CRC cell lines. We further obtained tissues from both the primary colon cancer and the metastasized liver of the same patient. Those tissues were also examined by qPCR and Western blot to assess the DAXX level. As shown in Figure 1C and D, in eight pairs of matched primary/metastatic cancer tissues, DAXX expression was significantly lower in the metastasized liver tissues than in the primary colon cancer tissues. In addition, immunohistochemistry results demonstrated that the DAXX level was lower in the metastatic sites than in the primary tumors (Figure 1E). These results suggest an association between the down-regulation of DAXX and CRC metastasis.

### Knockdown of DAXX promotes CRC metastasis *in vitro*

**Figure 2:**
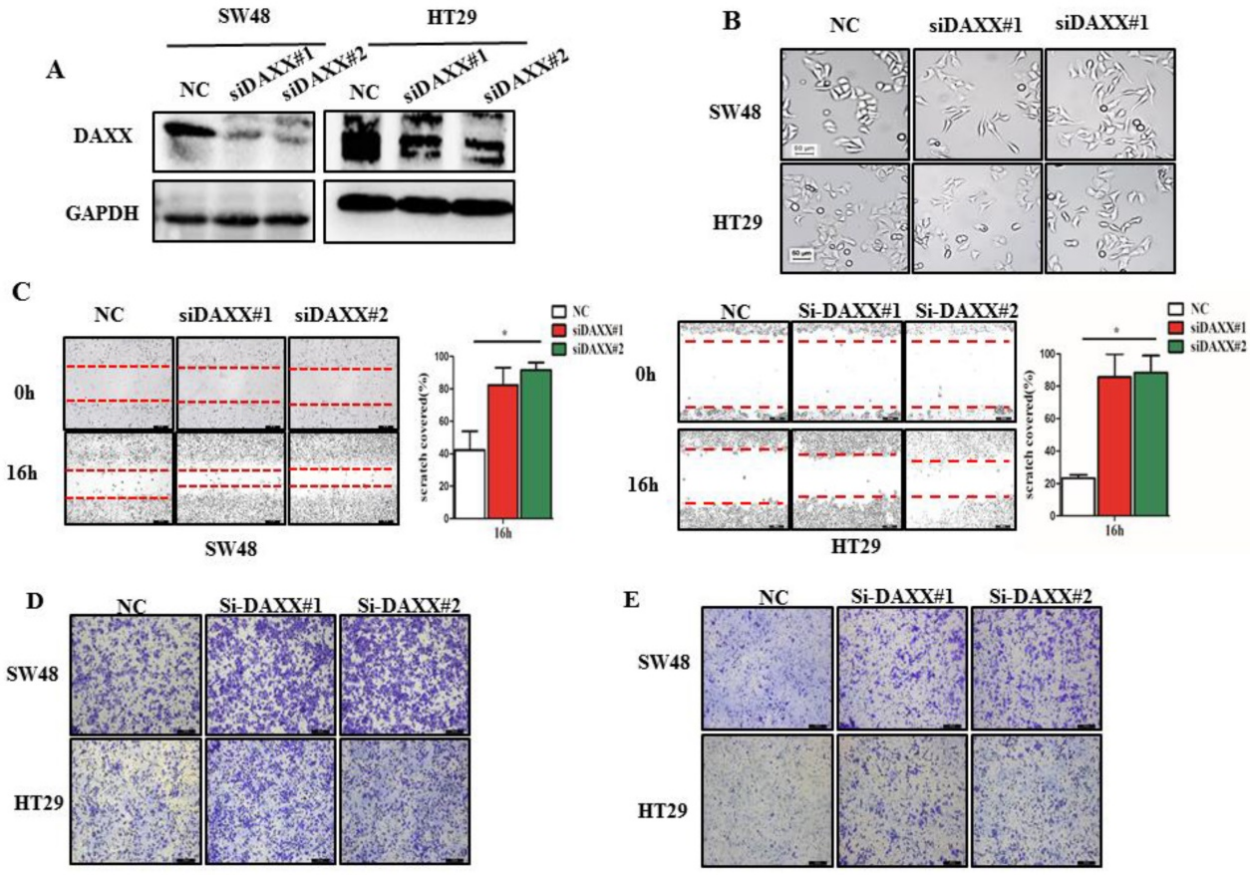
** DAXX knockdown and its association with CRC metastasis *in vitro*. (A)** Confirmation of the knockdown efficiency of transient transfection with siRNA in SW48 and HT29 cell lines by Western blot. **(B)** Morphological changes in SW48 and HT29 cell lines transfected with siRNA. **(C)** Cell motility in SW48 and in HT29 cell lines by wound-healing assay (*P<0.05). **(D)** Cell migration ability in SW48 and in HT29 cell lines by Transwell assay. **(E)** Cell invasion ability in SW48 and in HT29 cell lines by Transwell assay. (All scale bars: 50µm)

We first decreased the endogenous levels of the DAXX in HT29 and SW48 cell lines using two pairs of siRNA sequences. As shown in Figure 2A, the knockdown efficiency of transient transfection with siRNA of the two cell lines was assessed by Western blot. The results suggested that both pairs of siRNAs efficiently knocked-down DAXX expression. Figure 2B shows the transfected cells under a microscope. Cells transfected with siRNA-DAXX appeared to exhibit a spindle-shape and a loss of cell-cell contact, which are suggestive of EMT-related changes. Next, we evaluated the CRC cell motility by a wound-healing assay. As shown in Figure 2C, the motility test results demonstrated that knockdown of DAXX strongly increased the motility of both CRC cell lines compared to control cell lines (*P*=0.0187; *P*=0.0326). Meanwhile, cell migration and invasion were investigated using a Transwell assay. As shown in Figure 2D and E, DAXX silencing increased cell migration and invasion. These results indicated that the reduction of DAXX expression is associated with increased cell motility, migration, and invasion in CRC.

### Knockdown of DAXX decreases the expression of E-cadherin in CRC cell lines

**Figure 3:**
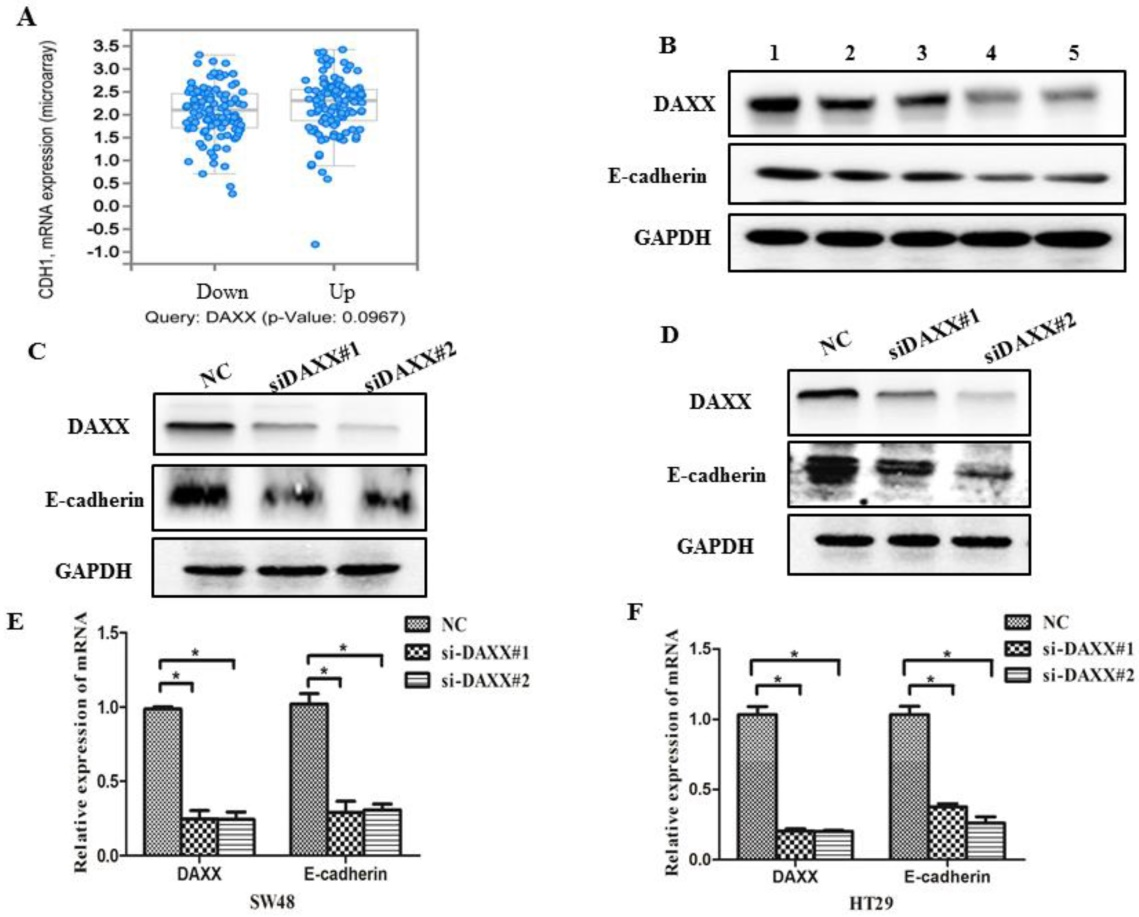
** DAXX knockdown and its association with E-cadherin expression in CRC cell lines. (A)** Association between mRNA expression of DAXX and E-cadherin according to a biological analysis database (http://www.cbioportal.org/). **(B)** Protein expression of DAXX and E-cadherin by Western blot. **(C) and (D)** Protein expression of E-cadherin in DAXX knockdown cells and control cell lines by Western blot. **(E) and (F)** mRNA expression of E-cadherin in DAXX knockdown cells and control cell lines by qPCR (*P<0.05).

As an initial step, we examined data from a biological analysis database (http://www.cbioportal.org/) and found an association between mRNA expression of DAXX and E-cadherin (Figure 3A). We then assessed the relationship between DAXX and E-cadherin in tumor tissues. As shown in Figure 3B, Western blot analysis revealed a positive correlation between DAXX and E-cadherin expression, indicating that E-cadherin may be involved in the reduction of DAXX-mediated metastasis. We next quantified E-cadherin level in DAXX knockdown cell lines and control cell lines. Western blot results showed that E-cadherin level was lower in DAXX knockdown cells than in control cell lines (Figure 3C and D). qPCR also showed that the expression of E-cadherin mRNA was significantly lower in DAXX knockdown cells than in control cell lines (Figure 3E and F; *P=*0.0415 and* P=*0.0152).

### Knockdown of DAXX enhances CRC metastasis by regulating E-cadherin

**Figure 4:**
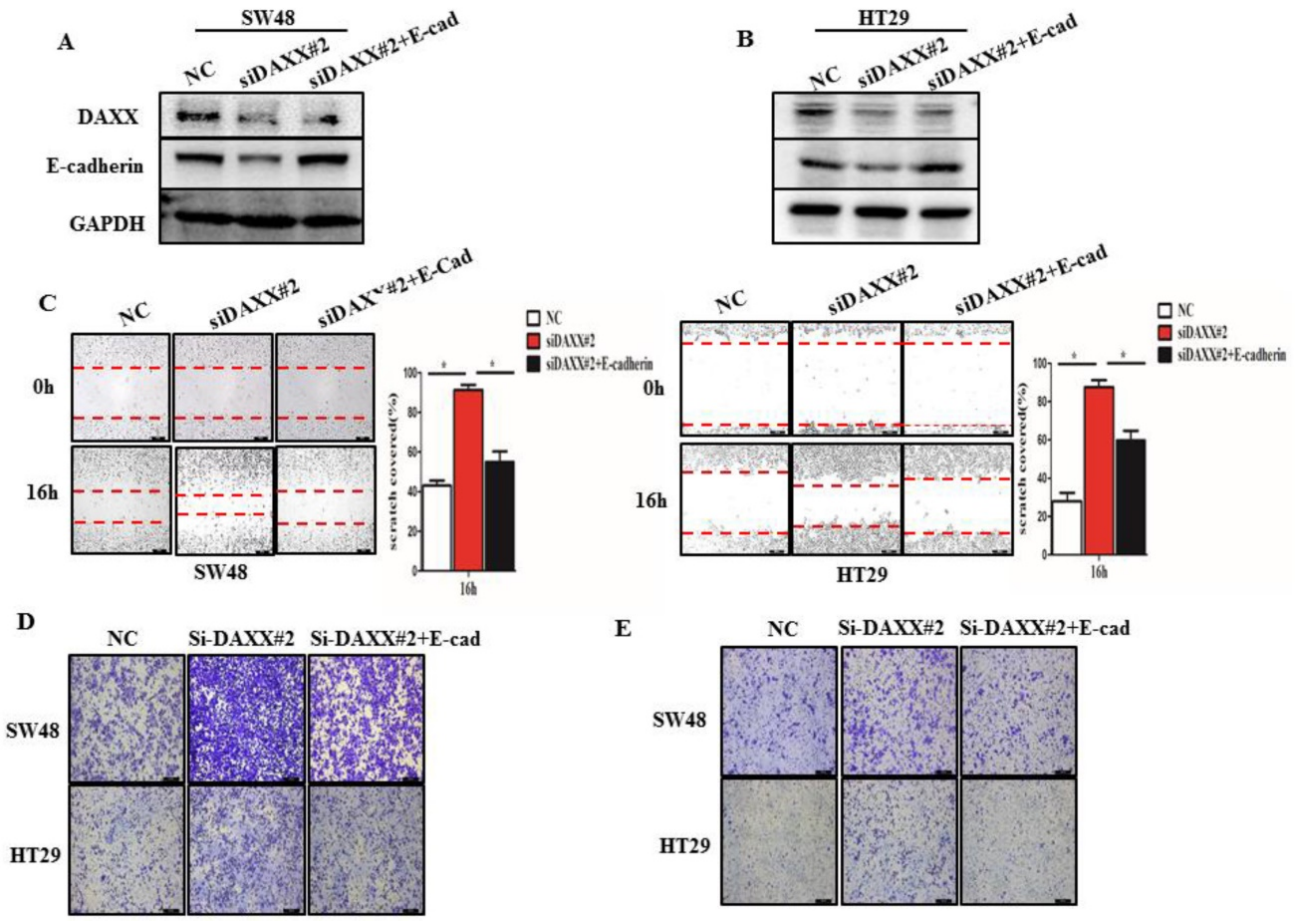
** The impact of DAXX and E-cadherin on CRC metastasis. (A) and (B)** Confirmation of the DAXX knockdown efficiency of transient transfection with siRNA and the overexpression efficiency of E-cadherin in SW48 and HT29 cell lines by Western blot. **(C)** The rescue effect of E-cadherin on cell motility in DAXX knockdown SW48 and HT29 cell lines by wound-healing assay (*P<0.05). **(D) and (E)**The rescue effect of E-cadherin on cell migration and invasion in DAXX knockdown SW48 and HT29 cell lines by Transwell assay (All scale bars: 50µm).

We further performed rescue experiments through transient co-transfection of cells with siRNA-DAXX and pcDNA3.1-E-cadherin plasmids. As shown in Figure 4A and B, Western blot confirmed that transient transfection with siRNA-DAXX and the pcDNA3.1-E-cadherin plasmids effectively knocked down DAXX and overexpressed E-cadherin, respectively. We also found that overexpression of E-cadherin can reverse the effects of DAXX knockdown on CRC motility using the wound-healing assay (Figure 4C; SW48: NC vs siDAXX, *P*=0.0278; siDAXX vs siDAXX+E-cadherin, *P=*0.0312; HT29: NC vs siDAXX, *P*=0.0179; siDAXX vs siDAXX+E-cadherin, *P=*0.0248). We further evaluated the cell migration and invasion capabilities using the Transwell assay. As shown in Figure 4D and E, we found that a decreased expression of DAXX can promote cell migration and invasion and that overexpression of E-cadherin can attenuate such an effect. These results suggest that the DAXX/E-cadherin pathway may be involved in CRC metastasis.

### DAXX interacts with ZEB1 and suppresses its inhibitory effect on promoter activity of E-cadherin

**Figure 5:**
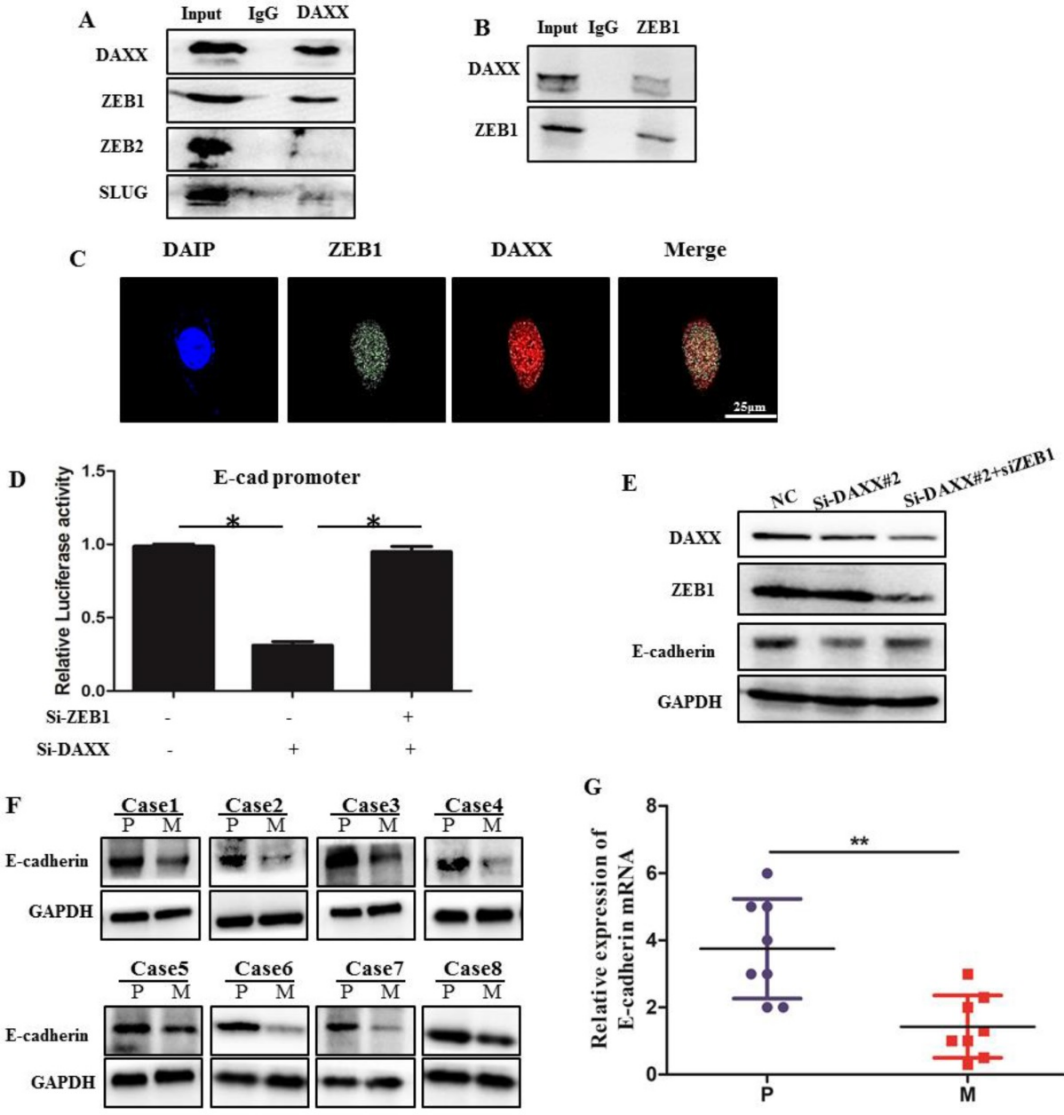
** The interaction of DAXX with ZEB1 and its role in E-cadherin expression. (A)** The interaction of DAXX with ZEB1, ZEB2, and SLUG by immunoprecipitation assay. **(B)** The interaction of ZEB1 with DAXX by immunoprecipitation assay. **(C)** The interaction of DAXX with ZEB1 by confocal experiments (Scale bar: 25μm). **(D)** The effect of DAXX- and ZEB1-knockdown on the E-cadherin promoter luciferase activity (*P<0.05). **(E)** The effect of DAXX-and ZEB1-knockdown on the protein expression of E-cadherin by Western blot. **(F)** Protein expression of E-cadherin in primary colon cancer tissues and metastasized liver tissues by Western blot (P: primary colon cancer tissue; M: metastasized liver tissues). **(G)** mRNA expression of E-cadherin in primary colon cancer tissues and metastasized liver tissues by qPCR (**P<0.05).

We performed qPCR and Western blot analysis on DAXX and E-cadherin mRNA and protein levels in transfected CRC cell lines. As shown in Figure 3, DAXX regulated the expression of E-cadherin at the transcriptional level. To verify whether DAXX regulates the expression of E-cadherin through direct interaction with certain transcription factors, we performed an immunoprecipitation assay using an anti-DAXX antibody and examined which transcription factors interact with DAXX. We found that ZEB1 bound strongly to DAXX, suggesting that DAXX may regulate E-cadherin by interacting with ZEB1. In addition, we did not find an interaction between DAXX and Slug or ZEB2 (Figure 5A and B). These results indicate that in colon cancer, DAXX preferentially interacts with ZEB1. Meanwhile, confocal experiments also showed that DAXX could directly interact with ZEB1 (Figure 5C). To further assess whether DAXX is involved in ZEB1-mediated E-cadherin repression, we transiently co-transfected the cells with pGL6 luciferase plasmids harboring the E-cadherin promoter fraction and siRNA-DAXX with/without siRNA-ZEB1. As shown in Figure 5D, knockdown of DAXX decreased the E-cadherin promoter luciferase activity, and this effect was reversed by co-transfection with siRNA-ZEB1 (*P*=0.0127; *P*=0.0237). In addition, Western blot showed that knockdown of DAXX led to a marked decrease in E-cadherin in SW48 cells which can be reversed by simultaneous knockdown of ZEB1 (Figure 5E). Also, as shown in Figure 5F and G, in eight pairs of matched primary/metastatic cancer tissues, E-cadherin expression was significantly lower in the metastasized liver tissues than in the primary colon cancer tissues. These results indicate that DAXX can modulate the activity of E-cadherin by blocking ZEB1-mediated transcriptional repression.

## Discussions

Distant metastasis in cancer is a multi-step process. During this process, cancer cell motility and invasion are believed to be critical for the migration of cells from the primary tumor to distant organs 6, 22, 23. Understanding how such a process is regulated at a molecular level can help identify novel targets for treatment, devise better strategies to prevent metastasis, as well as manage patients with metastasized diseases.

DAXX is a multi-functional protein involved in many physiological and pathological processes 24, 25. Increasing evidence shows that DAXX functions in tumor progression and its role is multi-faceted. It can serve as an oncogenic protein or as a suppressor in a cell-type-dependent manner. For example, recent studies have shown that DAXX is overexpressed in ovarian cancer, but suppressed in aggressive breast cancer 26, 27. Other studies suggested that DAXX is dysregulated and involved in pivotal events in the progression of several cancers 28. In colon cancer cells, DAXX was down-regulated, and the interaction between DAXX and Tcf4 was strongly associated with the proliferation of colon cancer cells, indicating that DAXX functions as a suppressor of tumor proliferation 13. In lung cancer, a report has shown that DAXX can inhibit cancer cell metastasis by directly blocking the HIF1a/HDAC1/Slug signaling pathway 11. However, the role and functions of DAXX in CRC remain poorly investigated. Our results demonstrated that DAXX is negatively correlated with tumor metastasis and that DAXX is down-regulated at the metastatic site compared to the primary lesion, implying that DAXX may act as a metastasis suppressor in CRC.

EMT is a vital process for cancer cell metastasis 23, 29, 30. To evaluate the role of DAXX in metastasis, we first knocked down DAXX in CRC cell lines. We found that knockdown of DAXX was sufficient to promote cancer migration and invasion *in vitro*. In addition, knockdown of DAXX dramatically induced morphological changes in the CRC cells from round-shaped to elongated and spindle-like. The latter is thought to have increased motility and invasiveness. These results suggest that DAXX actually plays a key role in CRC metastasis.

E-cadherin is a key factor in cell-cell adhesion. The destruction of E-cadherin is thought to be one of the first steps in metastasis, playing critical roles in tumor metastasis 21, 31-33. Recent reports have shown that a decrease in E-cadherin is strongly associated with metastasis and poor clinical prognosis and suggested that targeting E-cadherin could be a promising therapeutic approach for metastatic CRC 34. In this study, we showed that knockdown of DAXX can significantly decrease E-cadherin both at the transcriptional level and the translational level. Further, we found that overexpression of E-cadherin can reverse EMT and morphological changes induced by knockdown of DAXX. Our results provide evidence that DAXX regulates CRC metastasis through its effect on E-cadherin.

Recently, it has been reported that E-cadherin can be directly regulated by many transcription factors such as Slug, Snail, and Twist1, etc. 35. ZEB proteins, which are a family of transcription factors, have also been shown to directly bind to the promoter of E-cadherin and suppress its expression at the transcriptional level 21. DAXX can directly bind to many transcription factors and act as a potent transcriptional repressor 10. In this study, we attempted to investigate the relationship between DAXX and ZEB1 in the regulation of E-cadherin. We found that the decrease in E-cadherin induced by knockdown of DAXX can be reversed by knockdown of ZEB1. Our results suggest that DAXX regulates E-cadherin and metastasis in a ZEB1-dependent fashion in CRC. Further, both co-immunoprecipitation and confocal assays revealed that DAXX can directly bind to ZEB1 in the nucleus. Moreover, we showed that knockdown of DAXX decreases the E-cadherin promoter luciferase activity, which can be reversed by knockdown of ZEB1. These results demonstrated that DAXX can bind to the ZEB1 and block ZEB1-mediated inhibitory effect on promoter activity of E-cadherin in CRC.

In summary, we showed that DAXX expression decreased in metastatic lesions of CRC. We also demonstrated that DAXX modulated E-cadherin expression in CRC by suppressing ZEB1 activity, and the interaction between DAXX and ZEB1 is necessary for the regulation of E-cadherin expression. While our study requires further validation, our results suggest that reagents targeting the DAXX/ZEB1 pathway could help decrease cancer cell motility and invasiveness and thus prevent metastasis in CRC.

## Abbreviations

DAXX: death domain-associated protein
CRC: colorectal cancer
Tcf4: transcription factor 4
ZEB: zinc finger E-box binding homeobox
EMT: epithelial-mesenchymal transition
FBS: fetal bovine serum
pGL6: promoter-luciferase plasmids
qPCR: real-time reverse transcription-PCR
SD: standard deviation
